# Supplementary material for: Sympathetic Denervation Alters the Inflammatory Response of Resident Muscularis Macrophages upon Surgical Trauma and Ameliorates Postoperative Ileus in Mice
Source: Int J Mol Sci. 2021 Jun 26;22(13):6872. doi: 10.3390/ijms22136872 (PMC8268963; doi:10.3390/ijms22136872)
Supplement: Supplementary file 1 [file ijms-22-06872-s001.zip › ijms-1253822-supplementary.pdf]

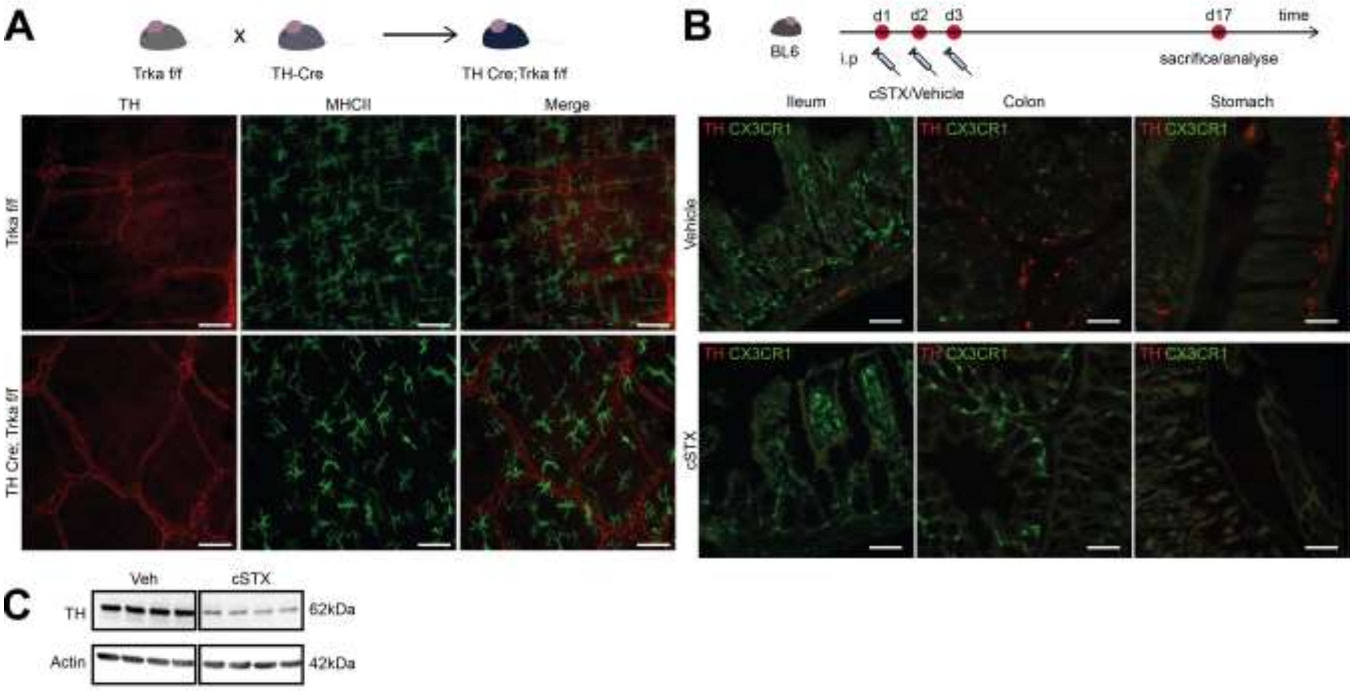

Supplemental figure S1: Analysis of the different sympathectomy models. (A) The experimental setting for the genetic (gSTX) model. TH immunofluorescence of ileal muscularis externa whole-mounts from TH-Cre;TrkAf/f mutant mice showed no difference in TH expression as compared to the control TrkAf/f mice. Scale bars, 100µm. (B) Representative histological sections of cSTX-treated ileum (bottom left), terminal colon (bottom middle), and stomach (bottom right) showing profound loss of TH^{+} sympathetic neurons as compared to vehicle-treated ileum (top left), terminal colon (top middle), and stomach (top right). Scale bars, 100µm. (C) Representative western blot image showing a reduction in TH protein bands in cSTX-treated mice compared to vehicle-treated mice.

Supplementary table S1: Primer sequences used for qPCR analysis.

Arg1	TTGGGTGGATGCTCACACTG<br>TTGCCCATGCAGATTCCC	Adr1a	TTGAAATTCGGGAAGAAGGA<br>GAGAAGAAAGCCGCCAAGAC
CD163	GTGCTGGATCTCCTGGTTGT<br>CGTTAGTGACAGCAGAGGCA	Adr1b	AGGCAGCTGTTGAAGTAGCC<br>TCTTCATCGCTCTCCCACTT
YM1	TCTCTACTCCTCAGAACCGTAAGA<br>GATGTTTGTCCTTAGGAGGGCTTC	Adr1d	ATTGAAGTAGCCCAGCCAGA<br>GCTGGTTCCCCTTTTTCTTC
IL6	AAGTCGGAGGCTTAATTACACATGT<br>CCATTGCACAACTCTTTTCTCATT	Adr2a	TCTGGTCGTTGATCTTGCAG<br>CATCTCGGCTGTCATCTCCT
IL1b	TTGACGGACCCCAAAAGATG<br>CAGGACAGCCCAGGTCAAAG	Adr2b	CAAAGCCTTTCATCTCCCTG<br>CTGGCCTCGACCTCACTAAG
TNFa	CTGAACTTCGGGGTGATCGG<br>GGCTTGTCACTCGAATTTTGAGA	Gapdh	GGGAAGCCCATCACCATCTT<br>GCCTCACCCCATTTGATGTT
Fizz1/<br>Retnla	SYBR green assay (<br>QT00254359)	AdrB1	AAGTCCAGAGCTCGCAGAAG<br>GCTGATCTGGTCATGGGATT
iNOS	AGGCCACATCGGATTTCACTTGCAA<br>(Taqman assay)	AdrB2	TAGCGATCCACTGCAATCAC<br>ATTTTGGCAACTTCTGGTGC
CSF1R	CTCTGCTGGTGCTACTGCTG<br>TTGCCTTCGTATCTCTCGATG	AdrB3	GGGGAAGGTAGAAGGAGACG<br>ACAGGAATGCCACTCCAATC
BMP2	AGACCACCGGCTGGAGAG<br>TTTTCCCACTCATCTCTGGAA	IL10	GATGCCCCAGGCAGAGAA<br>CACCCAGGGAATTCAAATGC
TH	AATGGGTTCCCAGGTTCC<br>AGCAGGATACCAAGCAGGC		
